# Expression Analysis of mir-21 and mir-221 in Cancerous Tissues from Iranian Patients with Gastric Cancer

**DOI:** 10.7508/ibj.2015.04.001

**Published:** 2015-10

**Authors:** Hosein Effatpanah, Reza Yadegarazari, Manoochehr Karami, Amir Majlesi, Nooshin Shabab, Massoud Saidijam

**Affiliations:** 1 *Research Center for Molecular Medicine, Hamadan University of Medical Sciences, Hamadan, Iran; *; 2 *Dept. of Genetics and Molecular Medicine, Hamadan University of Medical Sciences, Hamadan, Iran; *; 3 *Social Determinant of Health Research Center and Dept. of Biostatistics and Epidemiology, Hamadan University of Medical Sciences, Hamadan, Iran; *; 4 *Dept. of Gastrointestinal Disease, Beheshti Hospital of Hamadan University of Medical Sciences, Hamadan, Iran*

**Keywords:** MicroRNAs, Tumor markers, Stomach neoplasms

## Abstract

**Background:**

Early detection is a key to survival for gastric cancer. Molecular markers such as miRNA (microRNA) can have great importance in the early diagnosis of gastric cancer. Expression of miR-21 and miR-221 are deregulated in many types of human cancers. This study aimed to investigate the differences in miRNA expression patterns within the Iranian population.

**Methods:**

Total RNA was extracted from gastric cancer tissues and adjacent non-cancerous tissues from 32 patients. Expression levels of miR-21 and miR-221 were detected by Real time RT-PCR using a specific primer, with 5s rRNA as the internal reference gene.

**Results:**

Our data showed that the expression levels of miR-21 and miR-221 in gastric cancer samples were significantly higher than in paired non-cancerous samples (*P* < 0.05). The receiver operating characteristic (ROC) analyses yielded the area under the curve (AUC) values of 80.30 for miR-21 and 93.30 for miR-221, and combined ROC analysis revealed the highest AUC value of 96.90 in discriminating GC patients from healthy controls.

**Conclusion:**

It seems that miR-21 and miR-221 expression pattern in Iranian patients with gastric cancer are similar to any other population. Considering the increased expression level of two miRNAs in cancerous tissue compared to normal tissue as well as the area under ROC curve, miR-21 and miR-221 can be used for early detection of gastric cancer.

## INTRODUCTION

According to American Cancer Society report in 2011, gastric cancer is the fourth prevalent cancer worldwide and the third leading cause of cancer death in men and the fifth leading cause in women [1]. During the past 50 years, incidence of gastric cancer has decreased dramatically in western countries and Japan [2]. This trend is contrary to the recent reports from Iran, where the incidence of gastric cancer had been increasing during the past two decades [2]. According to Mehrabian *et al.* [3], the incidence is around 7300 cases per year, which is the most common cancer in men. Generally, because of delayed diagnosis, the five-year survival rate is only 20-25% [4]. In Iran, this rate is 12.8% [5]. Complete surgery resection is the most effective treatment for patients with early gastric cancer; however, the survival of patients with advanced gastric cancer is poor [6]. These data highlight the importance of early detection of gastric cancer. 

The previous studies have suggested a variety of protein serum tumor markers such as carcinoembryonic antigen and carbohydrate antigen 19-9 to facilitate early detection of gastric cancer [7, 8]. These tumor markers, however, are not highly sensitive and specific for early detection of cancer [9]. RNA makers, including mRNA and miRNA (microRNA) have been studied in common gastrointestinal cancers [10]. 

miRNAs are small, non-coding RNAs of 19-24 nucleotides in length that regulate the expression of target mRNAs. Therefore, to determine the function of miRNAs, the identification of target genes is essential. MiRNAs are involved in cancer-related processes such as cell differentiation and proliferation, apoptosis, metastasis, and death [11, 12]. Misregulation of specific miRNAs in human malignancies has been reported [13] though miRNAs could be used as molecular biomarkers for diagnosis of cancer and prediction of prognosis [14, 15]. According to recent studies, there have been some preliminary findings on the close correlation between miRNA expression and gastric cancer development [9, 14]. 

mMiRNA-21, as one of the most frequently studied oncomiRNAs, is a promising biomarker for the early detection of GC. While results of a meta-analysis on the potential diagnostic value of miR-21 for gastric cancer showed that miR-21 has potential diagnostic value with moderate sensitivity and specificity for GC [16], studies concerning the application of miR-21 in diagnosis of GC are still limited. During their meta analysis on potential diagnostic value of miR-21 for gastric cancer, Zeng *et al.* [16] included only five studies with a total of 251 GC patients and 184 control individuals.

mMiR-221 was also reported to be overexpressed in many malignancies, including gastric cancer [17, 18]. For example Liu *et al.* [17] indicated that miR-221 was upregulated in 88% of gastric cancer tissue samples compared with their paired adjacent non-tumor tissue samples. They also found that increased expression of miR-221 was associated with the advanced clinical stage of gastric cancer and poor survival.

The question of whether the expression of different miRNAs varies according to ethnic background has not been comprehensively and systematically evaluated. While there are some evidence on the relationship between miRNAs and gastric cancer, to best of our knowledge, there has been no previous work studying this issue in Iran.

In the current study, miR-21 and miR-221 as two potential oncogenic miRNAs were chosen to investigate the relationship between miRNA expression level and Iranian gastric cancer samples in a sample of gastric tumor tissue and adjacent non-tumour tissue.

## MATERIALS AND METHODS

***Patients and sampling. ***Gastric cancer tissues and corresponding normal tissues were obtained from 32 patients who had undergone a surgical gastric resection at the Cancer Institute, Tehran, Iran. The patients who had undergone radiation therapy, chemotherapy, or immunotherapy were excluded from the study. A summary of the pathological characteristics of the patients is given in Table 1. An informed consent was obtained from all patients, and the study was approved by the Medical Ethics Committee of the Cancer Institute. 

***RNA extraction. ***Total RNA was extracted with TRIzol reagent (Invitrogen, Carlsbad, CA, USA) following the manufacturer’s instructions. Briefly, tissue samples from each case were homogenized in 1 ml TRIzol reagent and then 0.2 ml of chloroform was added for phase separation. Following centrifugation at 12,000 ×g for 15 min, RNA was left exclusively in the aqueous phase and precipitated with 0.5 ml isopropanol. The RNA pellet was washed with 75% ethanol and dissolved in diethyl pyrocarbonate-treated water. The RNA quantity was measured with a Nano-Drop spectrophotometer (Bio-TeK, USA). The extracted RNAs were stored at -80ºC until cDNA synthesis. The quality of the RNAs was checked using agarose gel electrophoresis stained with SYBR Safe dye (Invitrogen, USA).

***cDNA synthesis.*** cDNA were synthesized using the ParsGenome’s miRNA amplification kit (ParsGenome Co., Iran). PCR primer pairs for miR-21 and miR-221 were obtained commercially from ParsGenome Co. (Iran). According to the manufacturer’s instructions, PCR primer was carried out in two main steps, including the addition of poly A and specific cDNA synthesis. In the first step, an appropriate amount of RNA (calculated with a nanodrop) was incubated with 2 µl 10× buffer, 2 µl ATP, 0.5 µl polyA polymerase enzyme, and diethyl pyrocarbonate-treated water at 37ºC for 10 min. Then in the second step, 5 µl polyadenylated RNA was mixed with 2 µl 5× buffer, 0.5 µl reverse transcriptase enzyme, 0.5 µl the linear primer, and 1 µl dNTPs. At the final point, the mixture was incubated in a thermal cycler at 42ºC for 60 min, followed by 85ºC for 5 s.

**Table 1:** Summary of characteristics of samples and miR-21/miR-221 expression

**Parameters**		**No (%)**	**miR-21 (** ***C*** **T)**	**miR-221 (** ***C*** **T)**
Age (years old)	<59	8 (25)	4.34 ± 1.01	5.40 ± 1.39
	≥59	24 (75)	4.10 ± 1.10	5.86 ± 2.26
				
Gender	males	26 (81)	4.32 ± 0.96	5.47 ± 1.92
	female	6 (19)	3.61 ± 1.43	6.96 ± 2.41
				
TNM stage	I	10 (31)	3.59 ± 1.14	5.18 ± 1.72
	II	22 (69)	4.45 ± 0.95	6.0 ± 2.19
				
Tumor size	≤5 cm	6 (19)	4.63 ± 0.88	6.11 ± 2.54
	>5 cm	26 (81)	4.08 ± 1.10	5.66 ± 1.99

TNM, tumor, lymph nodes, metastasis  stage

***Real time RT-PCR. ***Real time RT-PCR was performed using Bio-Rad thermal cycler (BioRad, USA). Reactions were carried out in a total of 20 µl, including 10 µl SYBR Green PCR Master Mix (PE Applied Biosystems, Foster City, CA, USA), 0.5 µl forward primer, 0.5 µl universal reverse primer, 8 µl nuclease-free water (CinnaGen, Iran), and 1 µl undiluted cDNA. All samples were processed in duplicate using Bio-Rad thermal cycler (USA) with the following cycling conditions: preliminary denaturation at 95°C for 5 min, followed by 45 cycles of denaturation at 95°C for 5 s, annealing at 62°C for 20 s, and elongation at 72°C for 30 s. 5s rRNA was chosen as the internal reference gene. ΔΔCT method was applied for analysis of real time RT-PCR results. For this purpose, mean of cycle threshold (CT) values for the reference gene and each of the miRNAs was calculated [19]. Threshold (CT value) is defined as the number of PCR cycles int which the fluorescent signal crosses the threshold. The difference of *CT* values of each miRNA and the reference gene was presented as Δ*CT*. ΔΔCT was calculated as the difference of Δ*CT *values between paired specimens. 2^-ΔΔ^^CT^ which represents the exponential value of Δ*CT *is considered as fold change difference in expression of two studied miRNAs between cancerous and non-cancerous tissues.

***Statistical analysis. ***Differences in mean between miRs (miR-21 and miR221) expression in the sample of gastric tumor tissue and adjacent non-tumor tissue as well as clinical parameters were analyzed using Student’s t-test. The correlation between ΔCt values of miR-21 and miR-221 was analyzed by Spearman's rank test. To determine to which extent the obtained −ΔCT value of miR-21 and miR-221 could efficiently separate different clinical subsettings, receiver operating characteristic (ROC) curves were drown, and the area under the ROC curve was calculated. Statistical analysis was performed with SPSS version 10 for Windows (SPSS Inc., Chicago, IL, USA). The difference was considered to be significant when the *P* value was less than 0.05.

## RESULTS

Associations between the content of miR-21 and miR-221 in gastric tissues and clinicopathological parameters were analyzed using the student’s *t*-test. The increased contents of miR-21 and miR-221 in gastric cancer were not associated with age (< 59 vs. ≥ 59), gender**,** tumor, lymph nodes, metastasis stage, or tumor size (≤5 cm vs. >5 cm). 

**Fig. 1:**
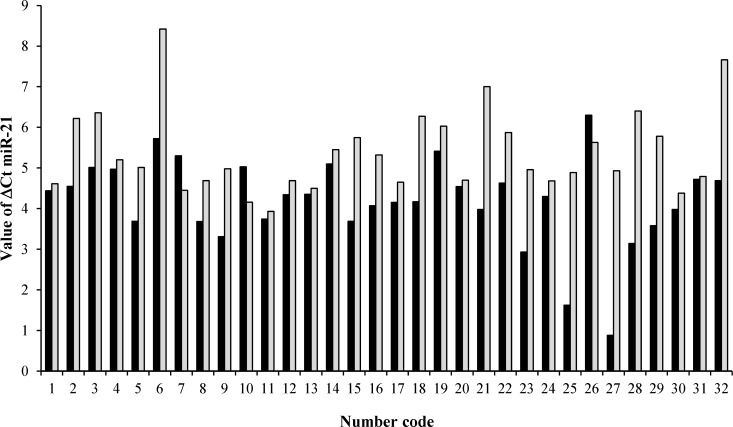
Ct values of miR-21 in human gastric tumor tissue and adjacent non-tumor tissue. The ΔCt range was from 0.88 to 6.30 in cancerous tissues, while it was from 3.93 to 8.42 in non-tumor tissues. Black and gray bars represent the expression level of miR-21 in patients and controls, respectively. Number code represents the number of patients

**Fig. 2:**
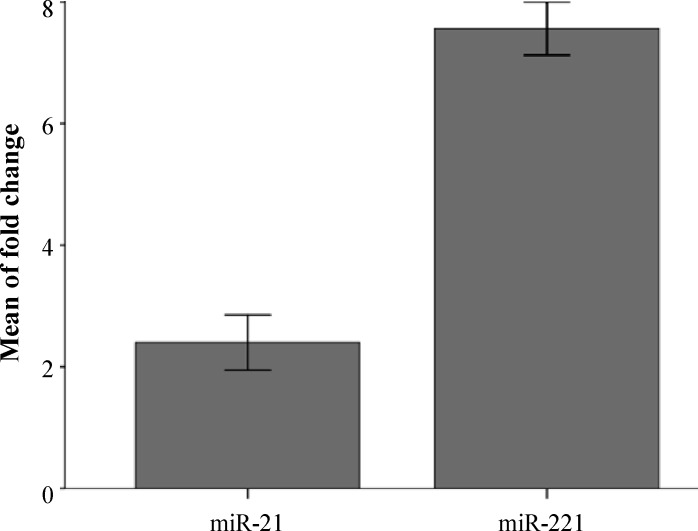
Fold change of the expression of the two miRNAs. The mean fold changes of miR-21 and miR-221 were 2.39 ± 2.56 and 7.5 ± 2.48, respectively

**Fig. 3:**
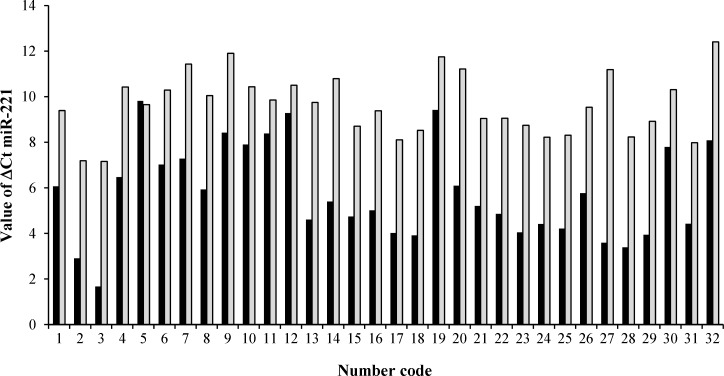
Ct values of miR-221 in human gastric tumor tissue and adjacent non-tumor tissue. The ΔCt range was from 1.67 to 9.81 in cancerous tissues, while it was from 7.16 to 12.40 in non-tumor tissues. Black and gray bars represent the expression level of miR-221 in patients and controls, respectively. Number code represents the number of patients

***miR-21 expression of paired cancerous and non-cancerous samples. ***The miR-21 was up-regulated in 90.62 % (29/32) of gastric tumor tissues compared with paired adjacent non-tumor tissue samples, with an average increase of 2.28 fold. (*P* = 0.012) (Figs. 1-3). The expression level and fold change of miR-21 in the normal and tumor samples from 32 patients are shown in Figures 1 and 2. The mean of Δ*C*T value for miR-21 in gastric tumor samples was 4.18 ± 1.07, as compared with 5.38 ± 1.01 in adjacent non-tumor tissue samples (*P *< 0.001).

***miR-221 expression of paired cancerous and non-cancerous samples.*** The miR-221 was up-regulated in 96.87% (31/32) of gastric tumor tissue compared with paired adjacent non-tumor tissue samples, with an average increase of 13.73 fold. (*P* = 0.012) (Figs. 1-3). The expression level and fold change of miR-221 in the normal and tumor samples from 32 patients are shown in Figures. 2 and 3. The mean ± SD *C*T value of miR-221 in gastric tumor samples was 5.75 ± 2.07, as compared with 9.63 ± 1.35 in adjacent non-tumor tissue samples (*P *< 0.001).

***Correlations between the expression of miR-21 and miR-221. ***ΔCt values of miR-21 and miR-221 were compared with each other by the spearman's rank test in cancerous or non-cancerous tissues, respectively. The results showed that there were significant correlations between the increasing of miR-21 and miR-221(r = 0.39, *P* = 0.02). 

**Fig. 4:**
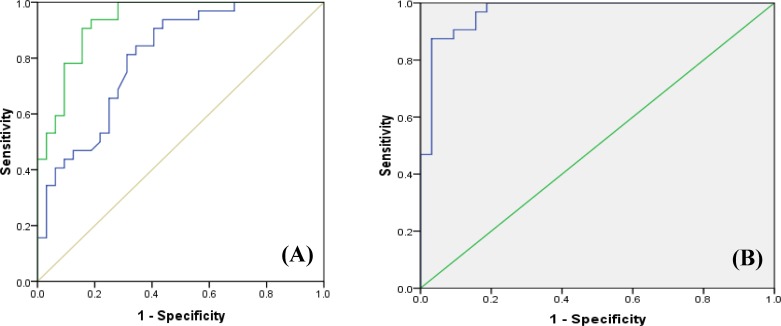
ROC curve analysis**. **ROC curve shows AUCs of miR-21 and miR-221 (A) as well as the combination of two miRs (B). The green and blue lines represent miR-21 and miR-221, respectively

***ROC curve analysis. ***ROC curve analysis was performed to evaluate the diagnostic value of miR-21 and miR-221 expression level in discriminating tumor and non-tumor states of the samples. ROC curve of the area under the curve (AUC) value for miR-21 was 80.3 (95% CI: 69.7-99) and for miR -221was 93.3 (95% CI 87.4-99.1). For evaluation of diagnosis value, the combination ROC curve analyses were calculated. Accordingly, the highest AUC value of 96.9 (95% CI: 93-1.07) which indicated that the combination signature has a strong potential diagnosis value for GC detection (Fig. 4).

## DISCUSSION

A review of the studies on the role of miR-21 and miR-221 suggests a significant overexpression of these two miRs in gastric cancer. Growing evidence suggests that miR-21 and miR-221 are involved in the regulation of cell proliferation and tumor progression [17, 20, 21]. However, because of limited knowledge, in recent years, association between altered miRNA expression and the development and progression of the gastric cancer has come to interest. The aim of this study was to investigate two important miRNAs (miR-21 and miR-221) expression levels in gastric cancer. Findings of the present study showed that the expression of miR-21 was significantly increased in gastric tumor compared with non-tumor tissues, which confirms the previous reports [14, 20, 22]. For example, Chan *et al*. [14] demonstrated that miR-21 was overexpressed in about 90% of the gastric cancer samples examined. Some previous studies detected high miR-21 expression levels in patients with clinicopathological features, including known prognostic factors [20, 22]. This result is expected since miR-21 is considered to act as an “oncomiR”, and high levels of miR-21 would be expected to correlate with a poor prognosis of patients. However, our data did not reveal an association between the levels of miR-21 and clinicopathological factors, including clinical stage of gastric cancer and tumor size. A limited number of samples in each studied group could be considered as an explanation for this result. Also, it can be attributed to our small sample sizes. However, a post-hoc power analysis indicated that study with this sample size had a 99% chance to detect a medium effect size when the type 1 error was 5%. This finding is consistent with that of a recent study by Chan and coworkers [14] who showed that patients with higher miR-21 expression did not have a worse prognosis. No differences in miR-21 expression were seen with regard to gender and age.

In the present study, miR-221 was also found to be over-expressed in gastric tumor tissue. On the other hand, similar results were also reported in other recent studies. For instance, Liu et al. [17] showed that miR-221 was up-regulated in 88% of gastric cancer tissue samples similar to miR-21. Furthermore, no correlation was observed between miR-221 expression level and age, gender, lymph node metastasis, or clinical stage of gastric cancer. Conversely, Fornari et al. [21] displayed that high expression of miR-221 is correlated significantly with advanced clinical stage, local invasive, and lymphatic metastasis. However in their study, no correlation was observed between miR-221 expression level and age or gender. Even in different individuals of the same race, variations in the expression of gene are possible [23]. Further studies with a larger cohort are needed to confirm these relationships.

Our results also revealed that there was a significant correlation between the fold changes of miR-21 and miR-221. ROC curve for miR-21 showed that the AUC value was nearly 0.8, indicating that the expression level of miR-21 might be used to diagnosis of gastric cancer. Combination ROC curve also showed that the AUC value was nearly 0.97, which indicated that the expression level of miR-21 and miR-221 together might be used for diagnosis of gastric cancer.

In conclusion, our study confirmed the over-expression of miR-21 and miR-221 in gastric tumor tissue samples and both of them can potentially serve as novel biomarkers for primary detection of gastric cancer in earlier stages. 

## References

[1] (2011). American Cancer Society: Global Cancer Facts & Figures.

[2] MalekzadehR, Derakhshan M (2009). H, Malekzadeh Z. Arch Iran Med.

[3] Mehrabian A, Esna-Ashari F, Zham H, Hadizadeh M, Bohlooli M, Khayamzadeh M (2010). Gastric cancer prevalence, according to survival data in iran (national study-2007). Iran J Public Health.

[4] Hartgrink HH, Jansen EP, van Grieken NC, van de Velde CJ (2009). Gastric cancer. Lancet.

[5] Movahedi M, Afsharfard A, Moradi A, Nasermoaddeli A, Khoshnevis J, Fattahi F (2009). Survival rate of gastric cancer in Iran. J Res Med Sc.

[6] Samadi F, Babaei M, Yazdanbod A, Fallah M, Nouraie M, Nasrollahzadeh D (2007). Survival rate of gastric and esophageal cancers in Ardabil province, North-West of Iran. Arch Iran Med.

[7] Nakane Y, Okamura S, Akehira K, Boku T, Okusa T, Tanaka K, et. al (1994). Correlation of preoperative carcinoembryonic antigen levels and prognosis of gastric cancer patients.

[8] Marrelli D, Pinto E, De Stefano A, Farnetani M, Garosi L, Roviello F (2001). Clinical utility of CEA, CA 19–9, and CA 72–4 in the follow-up of patients with resectable gastric cancer. Am J Surg.

[9] Tsujiura M, Ichikawa D, Komatsu S, Shiozaki A, Takeshita H, Kosuga T (2010). Circulating microRNAs in plasma of patients with gastric cancers. Br J Cancer.

[10] Yadegarazari R, Hassanzadeh T, Majlesi A, Keshvari A, Monsef Esfahani A, Tootoonchi A (2013). Improved real-time rt-PCR assays of two colorectal cancer peripheral blood mRNA biomarkers: a pilot study. Iran Biomed J.

[11] Krützfeldt J, Poy MN, Stoffel M (2006). Strategies to determine the biological function of microRNAs. Nat Genet.

[12] Bartel D (2004). MicroRNAs: genomics, biogenesis, mechanism, and function. Cell.

[13] Esquela-Kerscher A, Slack FJ (2006). Oncomirs—microRNAs with a role in cancer. Nat Rev Cancer.

[14] Chan SH, Wu CW, Li AF, Chi CW, Lin WC (2008). miR-21 microRNA expression in human gastric carcinomas and its clinical association. Anticancer Res.

[15] Xia L, Zhang D, Du R, Pan Y, Zhao L, Sun S (2008). miR-15b and miR-16 modulate multidrug resistance by targeting BCL2 in human gastric cancer cells. Int J Cancer.

[16] Zeng Z, Wang J, Zhao L, Hu P, Zhang H, Tang X (2013). Potential role of microRNA-21 in the diagnosis of gastric cancer: A Meta-Analysis. PLoS ONE.

[17] Liu K, Li G, Fan C, Diao Y, Wu B, Li J (2012). Increased expression of microRNA-221 in gastric cancer and its clinical significance. Int Med Res.

[18] Kim YK, Yu J, Han TS, Park SY, Namkoong B, Kim DH, et.al Functional links between clustered microRNAs: suppression of cell-cycle inhibitors by microRNA clusters in gastric cancer. Nucleic Acids Res.

[19] Livak KJ, Schmittgen TD (2001). Analysis of relative gene expression data using real-time quantitative PCR and the 2(-Delta Delta C(T)) Method. Methods.

[20] Zhang Z, Li Z, Gao C, Chen P, Chen J, Liu W, et.al (2008). miR-21 plays a pivotal role in gastric cancer pathogenesis and progression. Lab Invest.

[21] Fornari F, Gramantieri L, Ferracin M, Veronese A, Sabbioni S, Calin GA (2008). MiR-221 controls CDKN1C/p57 and CDKN1B/p27 expression in human hepatocellular carcinoma. Oncogene.

[22] Xu Y, Sun J, Xu J, Li Q, Guo Y, Zhang Q (2012). miR-21 is a promising novel biomarker for lymph node metastasis in patients with gastric cancer. Gastroenterol Res Pract.

[23] Rawlings-Goss, R, Campbell M, Tishkoff S (2014). Global population-specific variation in miRNA associated with cancer risk and clinical biomarkers. BMC Med Genomics.

